# How can accelerated development of bioenergy contribute to the future UK energy mix? Insights from a MARKAL modelling exercise

**DOI:** 10.1186/1754-6834-2-13

**Published:** 2009-07-03

**Authors:** Donna Clarke, Sophie Jablonski, Brighid Moran, Gabrial Anandarajah, Gail Taylor

**Affiliations:** 1 School of Biological Sciences, University of Southampton, Southampton, UK; 2 Imperial College Centre for Energy Policy and Technology, Mechanical Engineering Building, London, UK; 3 Institute for Energy Systems, School of Engineering, University of Edinburgh, Edinburgh, UK; 4 Geography Department, School of Social Science and Public Policy, King's College London, London, UK

## Abstract

**Background:**

This work explores the potential contribution of bioenergy technologies to 60% and 80% carbon reductions in the UK energy system by 2050, by outlining the potential for accelerated technological development of bioenergy chains. The investigation was based on insights from MARKAL modelling, detailed literature reviews and expert consultations. Due to the number and complexity of bioenergy pathways and technologies in the model, three chains and two underpinning technologies were selected for detailed investigation: (1) lignocellulosic hydrolysis for the production of bioethanol, (2) gasification technologies for heat and power, (3) fast pyrolysis of biomass for bio-oil production, (4) biotechnological advances for second generation bioenergy crops, and (5) the development of agro-machinery for growing and harvesting bioenergy crops. Detailed literature searches and expert consultations (looking inter alia at research and development needs and economic projections) led to the development of an 'accelerated' dataset of modelling parameters for each of the selected bioenergy pathways, which were included in five different scenario runs with UK-MARKAL (MED). The results of the 'accelerated runs' were compared with a low-carbon (LC-Core) scenario, which assesses the cheapest way to decarbonise the energy sector.

**Results:**

Bioenergy was deployed in larger quantities in the bioenergy accelerated technological development scenario compared with the LC-Core scenario. In the electricity sector, solid biomass was highly utilised for energy crop gasification, displacing some deployment of wind power, and nuclear and marine to a lesser extent. Solid biomass was also deployed for heat in the residential sector from 2040 in much higher quantities in the bioenergy accelerated technological development scenario compared with LC-Core. Although lignocellulosic ethanol increased, overall ethanol decreased in the transport sector in the bioenergy accelerated technological development scenario due to a reduction in ethanol produced from wheat.

**Conclusion:**

There is much potential for future deployment of bioenergy technologies to decarbonise the energy sector. However, future deployment is dependent on many different factors including investment and efforts towards research and development needs, carbon reduction targets and the ability to compete with other low carbon technologies as they become deployed. All bioenergy technologies should become increasingly more economically competitive with fossil-based technologies as feedstock costs and flexibility are reduced in line with technological advances.

## Background

### UK energy and climate change policy context

The UK Government states in the Energy White Paper 2007 [1] that the UK faces two long-term energy challenges, tackling climate change by reducing carbon dioxide emissions both within the UK and abroad and ensuring secure, clean and affordable energy. Following a recommendation by the new Committee on Climate Change (CCC) in 2008, the UK's CO_2 _reduction target was increased in the Climate Change Act from 60% to 80% below 1990 levels by 2050. Renewable energy is required as part of the future UK energy portfolio in order to meet CO_2 _reduction targets and improve energy security. An 80% reduction in CO_2 _emissions by 2050 coupled to the EU target of 15% supply of UK energy from renewables by 2020, represents ambitions that will require technology innovation, and better renewable deployment, as highlighted in the 2007 Stern Review [2].

The IEA's Technology Perspective [3] draws attention to the need for accelerated cost reductions and increased improvements in both new and existing energy technologies. The IEA recognises this will take a large commitment to research, development and demonstration (RD&D) from the private and public sectors.

### Bioenergy technologies and their potential contribution to the UK's carbon ambitions

Bioenergy is one of the most prominent options to reduce CO_2 _emissions, if it is produced in a sustainable way, and currently contributes approximately 80% of renewable power production in the UK [4]. Most of this, however, is from methane associated with landfill. Which bioenergy technologies are deployed in the future will depend partly on national and international policies and support, but the move towards a low carbon economy, with a price associated with carbon, is also likely to stimulate technology development for renewables [3]. It is predicted that near future investments in European countries are likely to focus on renewables, among other energy sectors, with an emphasis on biomass [3].

Bioenergy technologies are numerous and varied, incorporating many feedstocks, methods of conversion and supply routes to end products and end uses. In addition, bioenergy technologies can be found at all levels of maturity ranging from well established proven technologies, to new technologies that are in the research and development (R&D) phase. As a consequence, it is not possible to characterise the maturity of the bioenergy field as a whole. This can also partially explain why bioenergy research remains extensive and cross-disciplinary. Finally, such multi-disciplinarity and complexity means that it is not yet well understood how influential technological development will be for bioenergy's contribution to future low carbon energy systems.

### The objective

The main objective of this paper is to explore the possible contribution of emerging bioenergy technology to the UK energy system by 2050, by outlining the potential for accelerated technology development (ATD) of bioenergy systems in the UK. We aim to gain insight into how bioenergy technologies may contribute to meeting the 80% carbon reduction targets for a low carbon energy system in the UK, by using a MARKAL model complemented by relevant qualitative storylines highlighting key factors underpinning modelled technological development.

### The UK Energy Research Centre Energy 2050 project and modelling context

The UK Energy Research Centre (UKERC) Energy 2050 project focuses on how the UK energy system may move towards a resilient, low-carbon system by 2050, while providing energy security [5]. By using a set of four core scenarios ('Reference', 'Low Carbon', 'Resilient' and 'Low Carbon Resilient'), and variant scenarios (such as the 'Accelerated Technology Development' scenarios), the project incorporates the policy, environmental and social aspects which may lead to possible future UK energy systems [5]. As part of the UKERC Energy 2050 project, UK-MARKAL (MED) was used to explore the possible contribution of accelerated technology development to the uptake of bioenergy-based technologies in the UK energy system by 2050. MARKAL is a technology-rich, least cost optimisation model which has been used in the past to inform energy policy [6]. A fuller description of the UK MARKAL Energy System Model is described by Strachan *et al*. [7] and the way bioenergy is modelled by Jablonski *et al*. [6].

**Figure 1:**
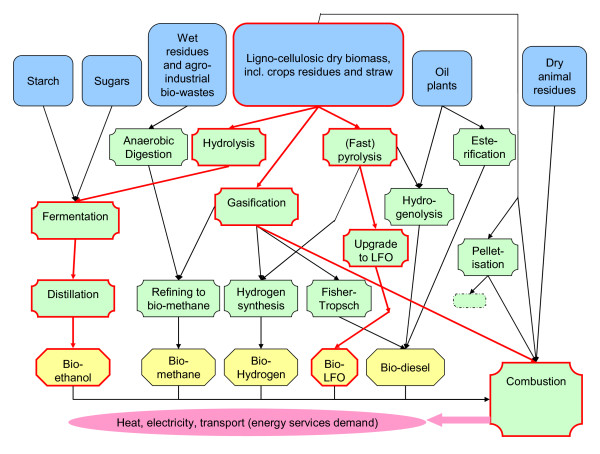
**UK-MARKAL (MED) simplified bioenergy chains, with gasification, lignocellulosic bioethanol and fast pyrolysis bio-oil highlighted**.

Bioenergy pathways are represented in MARKAL by more than 100 directly relevant technologies in the different modules of the model (and more than 200 indirectly relevant ones). Figure 1 provides a simplified representation of the bioenergy conversion pathways in MARKAL (highlighting the lignocellulosic ethanol, gasification and fast pyrolysis pathways).

## Methods

### Overview of the methodological framework

**Figure 2:**
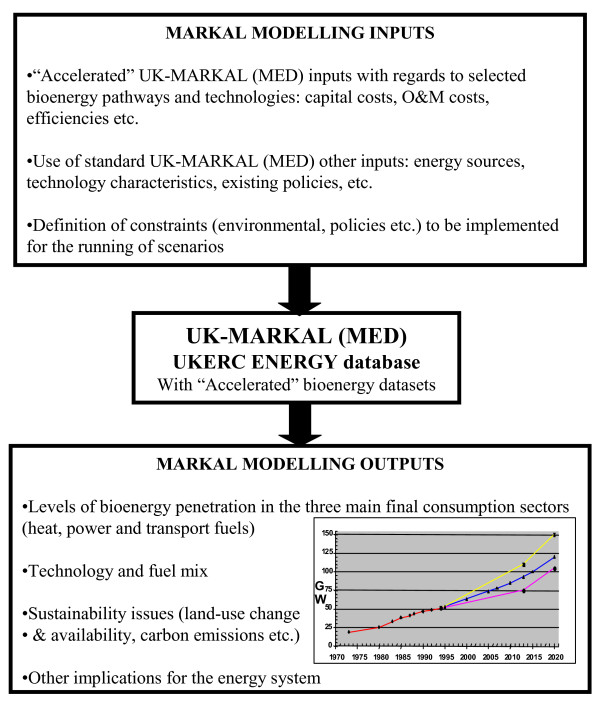
**Methodology framework used to assess the potential accelerated technology development (ATD) of bioenergy chains**.

The methodology is summarised in Figure 2. The research focuses on the modelling of ATD in MARKAL, its qualitative characterisation for the case of the UK, and the possible implications of such technological development for the UK energy system. It was essential to the ATD exercise that all scenario runs were underpinned by the development of qualitative information of R&D needs and potential.

### Criteria used for (and results of) bioenergy technology selection

In order to explore accelerated technology development of bioenergy, this research focused specifically on accelerated *development *of the bioenergy technology field, not accelerated *deployment*. Bioenergy is a wide field, representing a large number of chains with many feedstocks, conversions and supply routes that feed into heat, power and liquid biofuels in the UK [8] and it was not possible to study all pathways in detail. To focus on a limited number of bioenergy chains, a set of technologies were chosen which had, (i) the greatest potential for technology development and commercial deployment; (ii) were represented in the MARKAL model; and (iii) had the potential to be environmentally sustainable in the long term, with the focus on bioenergy pathways with the potential to be technically available, assuming that no additional pressures on biodiversity, soil or water resources are exerted compared with a development without bioenergy production, in line with the 2006 European Environment Agency report [9]. Based on these criteria, the chains and technologies selected for an extensive exploration of the potential for ATD of bioenergy systems in the UK included (Table 1) the following.

**Table 1:** Rationale used to develop the accelerated technology development (ATD) scenarios for the five technologies.

**Technology cluster**	**Rationale for ATD**	**Key changes in datasets**	**Key references**
Lignocellulosic conversion to ethanol	Improvement in feedstock quality Better conversion technologies Cheaper enzymes	Availability Efficiency Investment costs O&M costs	R. Slade (unpublished data); [28]

Gasification of solid biomass (energy crops)	Increased fuel flexibility Cheaper feedstocks Cleaner gas production	Availability Efficiency Capital costs Fixed O&M costs	J. Brammer (unpublished data); [25,29]

Fast pyrolysis	Better grade/cleaner oil Improved feedstocks Increased fuel flexibility Cost reductions associated with the above	Capital costs Efficiency O&M costs	J. Brammer, J. Rogers (unpublished data); [22]

Bioengineering of energy plants	Increased yield Increased stress tolerance, and disease and pest resistance Increased nitrogen and water efficiency	Improved yield and energy content of energy plant	[12,20,24,30-34]

Agro-machinery of growing and harvesting energy crops	Improved establishment on marginal/idle land Increased efficiency at picking up cut crops Improvement in irrigation systems Improved site preparation methods and weed elimination methods	- incorporated into bioengineering of energy plants, as part of resource costs	C. Panoutsou (unpublished data) [20,35]

#### The conversion of lignocellulosic second generation feedstock to bioethanol

This was chosen because considerable technological advances are likely [10] and because liquid biofuels provide one of the few options for fossil fuel replacement in the short to medium term, with the potential to offer both greenhouse gas savings and energy security [11].

#### Gasification of solid biomass

Although this is not a new technology, gasification was selected as it a process working towards deployment at demonstration and commercial scale and technology development is possible [12]. This modelling exercise focused on gasification of dedicated energy crops used directly for electricity generation, rather than on technologies where biomass is first converted into biogas and upgraded into bio-methane before being transformed into electricity through a (natural) gas turbine.

#### Fast pyrolysis of biomass for the production of bio-oil

This is a technology largely at the early commercial stage; however, the production of transport fuels via fast pyrolysis is still in the R&D stage with potential for further advances [13]. This exercise focused on the pyrolysis of wood to produce bio-oil. Within the model, this bio-oil can go to three possible pathways: further pyrolysis to hydrogen, leading to the transport module; upgrade of pyrolysis oil into light fuel oil, which goes to the industry, residential or services sector, or upgrade of pyrolysis oil into bio-diesel, which goes to electricity production or transport.

#### Bioengineering of energy crops

This represents one of the underpinning technologies, as feedstock prices underpin many of the costs associated with bioenergy chains. The focus was on improvements through better breeding to advance dedicated second generation energy grasses and trees, not food crops. We focused on non-GM crops and domestic (UK) crops. The focus on domestic crops only was to reflect long-term environmental sustainability goals.

#### Agro-machinery for growing and harvesting energy crops

The other underpinning technological improvement selected was the potential for improved machinery for growing and harvesting dedicated bioenergy crops. Good site preparation and weed elimination are highly influential on the performance of many energy crops, and improvements in these areas are important. There are also crop losses associated with inefficient harvesting/picking up of cut energy crops for example, which need to be addressed. Improvement in both agro-machinery and bioengineering of energy crops are likely to affect learning curves and supply costs for multiple bioenergy chains [14].

### Data collection for the ATD Bioenergy for UK-MARKAL (MED) modelling

The current status of each of the five chains and their potential for acceleration were assessed using data collected on both a qualitative (R&D needs, UK and international research efforts, and policy considerations) and a quantitative basis. This information was obtained from published literature, government reports and expert consultation.

Qualitative information for the scenarios was used to estimate possible future technology developments until 2050, through processes such as gradual changes, step changes and innovation, as well as gaining an understanding of the possible milestones for each technology.

The ATD bioenergy quantitative dataset was compiled for each of the five technologies using optimistic figures from literature searches and expert consultation to represent accelerated technology development from 2000 to 2050. The information available varied widely between the five technologies chosen. Accordingly, the assumptions and the process by which the data in the literature was used to determine the accelerated dataset are described below for each technology separately. The data collected consisted of figures on capital cost, operating and maintenance cost, technical efficiency, defined as the ratio between the useful output of energy conversion to the input, annual availability, defined as the share of the installed capacity that is used during a year (average share of the year), plant lifetime in the case of electricity generation and for biotechnological advances for second generation bioenergy crops, energy content and yield. Acceleration was represented through reducing costs, increasing efficiencies and including earlier availability for the technologies, in line with the literature and expert consultation. Since the UK-MARKAL database costs are in pounds sterling (GBP), all cost data were converted to GBP on a year 2000 basis.

### Overview of the modelled bioenergy ATD scenarios

Once the data on accelerated technology development for selected bioenergy pathways was compiled, it was included in the modelling of five different 'accelerated' scenarios (Table 2) to explore how bioenergy technologies may penetrate the UK energy market if technology development is accelerated. These accelerated scenarios were built around the UKERC Energy 2050 project scenarios. The scenarios were produced as a 'what-if' exercise to determine how accelerated technology development could influence the future energy mix to reflect possible technology improvements through present and future R&D efforts and, therefore, should not be taken as being predictive.

**Table 2:** Description of the five scenarios run as part of the accelerated technology development (ATD) scenarios.

**Scenario**	**Description**
ATD Bioenergy	All five bioenergy technologies were accelerated together. No acceleration of any other technologies

LC-Acctech (60%) without fuel cells	All technologies (wind, marine, bioenergy, solar PV, coal CCS, and nuclear) accelerated together to achieve a 60% reduction in carbon emissions by 2050

LC-Acctech (80%) without fuel cells	All technologies (wind, marine, bioenergy, solar PV, coal CCS, and nuclear) accelerated together to achieve an 80% reduction in carbon emissions by 2050

LC-Acctech (60%) with fuel cells	All technologies (wind, marine, bioenergy, solar PV, coal CCS, nuclear and fuel cells) accelerated together to achieve a 60% reduction in carbon emissions by 2050

LC-Acctech (80%) with fuel cells	All technologies (wind, marine, bioenergy, solar PV, coal CCS, nuclear and fuel cells) accelerated together to achieve an 80% reduction in carbon emissions by 2050

This paper focuses on the contribution of bioenergy to decarbonising the energy system; however, further exploration of the other technology scenarios can be found in the forthcoming ATD report from UKERC Energy 2050 [15].

### Application to selected bioenergy chains in the UK

#### The Accelerated Bioenergy scenario; how acceleration was modelled

The representation of technological development in MARKAL has been done by the introduction of technologies' vintages (that is, similar technologies available at different times) with differing parameters corresponding to technological evolution, to represent learning effects or other advances in technology development. These parameters include capital cost, efficiency, operating and maintenance (O&M) costs, both fixed and variable, and where appropriate, availability, contribution to peak load, and plant life time.

It is important to have an understanding of the R&D needs of a technology pathway when assessing its potential for accelerated technology development. Understanding the major hurdles to development and deployment is also critical when considering the likelihood of technology breakthroughs and step changes within a technology pathway.

Bioenergy is diverse and flexible, covering many feedstock resources, conversion pathways and outputs [16]. As such, there are unique R&D needs for each of these different elements of the bioenergy chain. There are, however, two critical areas of R&D for the bioenergy field as a whole: improving crops and improving conversion technologies [4,16].

The development of new dedicated bioenergy crops for feedstocks is one of the most fundamental R&D needs for bioenergy, as this underpins the development and cost of many bioenergy conversion technologies [4]. The UKERC Research Atlas for bioenergy [16] identifies research challenges for bioenergy over the next 5 years including the development and delivery of new cultivars from past and current research and breeding of dedicated energy crops. In the next 10 years, there is a need to improve the total yield and develop new genotypes for a range of bioenergy crops, including oil seed crops, aquatic biomass, woody lignocellulose and grasses. R&D needs for second generation energy crops include new genotypes and selective breeding to increase yields and system efficiencies, such as improving stress tolerance, disease and pest resistance, increased photosynthetic, nitrogen and water use efficiency and increased biomass production (Table 1) [4,16-19]. It is likely that a 30% increase on current yield will be possible over the next 10 to 15 years, using traditional breeding and selection [4]. Advances in biotechnology of second generation bioenergy crops will additionally help to make feedstocks cheaper, which is important for technologies such as the production of lignocellulosic ethanol, gasification and fast pyrolysis, which require cheaper feedstocks if overall costs are to be reduced (J. Brammer, J. Rogers, unpublished data).

Improved establishment of dedicated bioenergy crops on marginal and idle land, as recommended by the Gallagher Review [13] would also help to reduce land competition and avoid displacement of food crops, possibly increasing the social acceptance of bioenergy. This could increase the land area available to produce energy crops.

Advances in site preparation, weed elimination [16] and improvements in the agro-machinery used to grow and harvest dedicated bioenergy crops is additionally needed (C. Panoutsou, unpublished data). Improvements include increasing not only the engine/fuel efficiency of agro-machines, but also their efficiency at picking up the harvested crop to reduce crop losses [20], integrating different crop types with different harvest times, and producing better irrigation systems which can cope with particles contained in recycled water (C. Panoutsou, unpublished data).

Technical improvements in existing conversion technologies such as gasification, and novel conversion technologies like fast pyrolysis, are also needed. R&D needs for both of these technologies include increasing conversion efficiency, reducing overall technology costs, increasing fuel flexibility so that a variety of new energy crops can be utilised as feedstocks and improving product quality through gas cleaning in gasification and producing cleaner bio-oil from fast pyrolysis [11,18,21-25]. All of these improvements will push gasification and fast pyrolysis technologies towards commercial deployment through increased economic viability, via the ability to scale-up.

The economic competitiveness of biofuels compared with conventional fuels is a key barrier in the deployment of biomass in the transport sector [26]. In order to stimulate a more efficient and sustainable conversion from lignocellulosic biomass to ethanol, key R&D needs include the improvement of feedstock flexibility and quality to enable easier breakdown of cell walls, in particular less lignin, but also development of *in-situ *enzyme systems for wall disassembly [10,26]. Better conversion technologies, with less pre-processing and enzymes with lower costs are also required [10] to make lignocellulosic ethanol more economically competitive with conventional fuel.

Table 1 lists the rationale, and parameters which were changed to represent accelerated technology development for each of the five bioenergy chains, while Table 3 compares how accelerated technological development was represented in UK-MARKAL (MED) for the ATD scenarios to reflect possible technology improvements through present and future R&D efforts. The changes between the LC-Core scenario and the ATD scenarios are outlined in more detail below. Although these datasets are based on extensive literature reviews and expert consultant, it is important to highlight that these cost reductions are very uncertain and all figures should be taken with caution.

**Table 3:** LC-Core and ATD Bioenergy data for UK-MARKAL.

**Technology**	**Year**	**Capital cost** **(GBP.GJ^-1^)**		**Annual availability (%)**		**Efficiency (%)**		**O&M cost (GBP.GJ^-1^)**	
		
		**Core**	**ATD**	**Core**	**ATD**	**Core**	**ATD**	**Core**	**ATD**
**Lignocellulosic ethanol**	2010	23	23	100	90	100	90	2	1
	
	2050	23	14	100	90	100	90	2	0.5

**Technology**	**Year**	**Capital cost** **(GBP.kW_e_^-1^)**		**Annual availability (%)**		**Efficiency (%)**		**Fixed O&M costs (GBP.(kW.yr)^-1^)**	
		
		**Core**	**ATD**	**Core**	**ATD**	**Core**	**ATD**	**Core**	**ATD**

**Gasification**	2000	2,200	2,200	83	85	32	32	66	66
	
	2050	1,673	700	83	89	44	50	66	30

**Technology**		**Year**	**Capital cost** **(GBP.GJ^-1^)**		**Efficiency (%)**			**Variable O&M costs** **(GBP.GJ^-1^)**	
			
			**Core**	**ATD**	**Core**		**ATD**	**Core**	**ATD**

**Fast pyrolysis**		2000	32.4	32.4	90		90	3	3
		2050	32.4	25.6	90		90	3	1

**Chain/technology**			**Year**	**Capital cost** **(GBP.GJ^-1^)**		**Energy content (GJ.t^-1^)**		**Yield (ha.yr^-1^)**	
				
				**Core**	**ATD**	**Core**	**ATD**	**Core**	**ATD**

**Bioengineering of energy crops (including agro-machinery)**			2000	3.61	3.61	-	15	-	12
			2050	3.61	1.45	-	15	-	24

All cost data are expressed in GBP on a year 2000 basis. Agro-machinery costs are assumed to be embedded in energy crop costs (resources module).

### Lignocellulosic ethanol

Major technology improvements and accelerated development which will reduce overall costs and increase efficiency of lignocellulosic conversion to ethanol are expected from a combination of improvements in feedstock quality, with reduced lignin for better breakdown of cell walls, cheaper enzymes and more efficient conversion technologies, which require less pre-processing, plus an improvement in the fermentation process (Table 1).

Parameters changed within MARKAL included capital costs and O&M costs. All other costs associated with this technology were kept the same as the core scenario. The parameters used to model lignocellulosic ethanol conversion were changed as follows to represent accelerated development (Table 3).

Lignocellulosic ethanol is available in the model from 2010. In the core scenario, it is modelled with an annual availability of 100%, which was reduced slightly to 90% in the accelerated scenario.

In the core scenario the efficiency is modelled as 90%. Although this figure is too high, with expected efficiencies to be around 30 to 40% (R. Slade, unpublished data), the efficiency was kept at 90% in order to keep it comparable to the non-accelerated scenario and to avoid any drastic model 'deceleration', as this would be contrary to the aim of the exercise. Laser *et al*. [27] suggested that mature cellulosic ethanol technology could reach efficiencies of 68% in combination with GTCC. The figures used to represent accelerated development, therefore, should be taken with caution, and are used as an illustration only, not a prediction of technical improvement.

The investment costs of lignocellulosic ethanol conversion in the core scenario are 23 GBP.GJ^-1^, which is in line with recent US Department of Energy (DOE) research [28]. In the accelerated scenario, investment costs were reduced following the trend indicated by DOE to reach 14 GBP.GJ^-1 ^by 2050. These changes in investment costs are likely to occur as economies of scale are obtained when it is possible to construct larger plants (in line with increased investors' trust, better access to capital etc.) (R. Slade, unpublished data).

For the variable O&M costs, the data used in the core scenario (1.9 GBP.GJ^-1^) seems too high. In the accelerated scenario, O&M costs were reduced to 5% of the investment costs in 2000 and 2010, and to 2% of the total investment costs by 2050. These percentages are in line with expert analysis and estimates on expected development of the technology (R. Slade, unpublished data).

### Gasification

The basis for acceleration of gasification technologies comes from improved production of cleaner gases, and cheaper feedstock coupled with increased fuel flexibility, which will help to reduce overall costs of gasification and increase the feasibility of scaling-up (Table 1). Currently feedstock accounts for around one third of the costs associated with gasification, and in combination with the above, an increase in feedstock flexibility would greatly help to reduce the over-costs of this technology (J. Rogers, unpublished data).

The main figures used for the cost assessment of gasification were obtained from the Department of Trade and Industry (DTI) [29] (a forerunner of the Department for Business, Enterprise and Regulatory Reform (BERR)) in the UK and the National Renewable Energy Laboratory (NREL) Power Energy Technologies Databook in the US [25], and figures used are therefore based on the assumptions used within these reports. When unavailable, project costs to 2050 have been extrapolated via cost curves from the optimistic figures given in the energy crop gasification literature.

Energy crop gasification is represented in MARKAL by existing gasification (2000) and a number of technology 'vintages' in the model, including one at 2010, 2020, 2030 and 2040. The following data were used for the ATD gasification scenario (Table 3).

The annual availability in 2000 was modelled at 83% in the core scenario. For the accelerated technology scenario, this was increased to 85% in 2000 in line with the DTI economics report [29], and increased gradually to 89% by 2050.

The efficiency data for 2000 was kept at the same starting point for both scenarios (32%), but was increased to 47% in 2010 and 2020, and 50% in 2030 and 2040 in the accelerated scenario [29].

Capital costs for the accelerated scenario were kept the same in 2000 as in the core scenario at 2,200 GBP.kW_e_^-1^, reducing to 1,450 GBP.kW_e_^-1 ^in 2010, and 700 GBP.kW_e_^-1 ^by 2020 as reported in the DTI [29] and could occur from a combination of factors. This cost was then assumed to level off after 2020 based on technology assumptions from the literature.

Under the accelerated technology scenario, the fixed O&M costs in 2000 were kept at 66 GBP.(kW_e_.yr)^-1^, decreasing to 51.5 GBP.(kW_e_.yr)^-1 ^in 2010 and to 30 GBP.(kW_e_.yr)^-1 ^by 2030 to 2040 to be consistent with figures reported in the DTI ecomomics report and NREL powerbook [25,29].

### Fast pyrolysis

The accelerated development of fast pyrolysis and therefore the ATD figures are based on producing cleaner bio-oil, improving processing and increasing the fuel flexibility of fast pyrolysis (Table 1). Although there were a number of studies that examined the economics of fast pyrolysis, many were unsuitable because they either measured the cost of the bio-oil production rather than the capital cost, or because they did not present enough information to convert their cost figures into a comparable capital cost as found in the model. The one study that was applicable was from the DTI [24]. This study presented a low, medium, and high levelised capital cost estimate from 2005 to 2020 in GBP.MWh^-1^.

The DTI's medium scenario was in line with the LC-Core scenario data. In order to show the potential for acceleration of fast pyrolysis, in keeping with the discussions with experts (J. Rogers, J. Brammer, unpublished data) and the literature available, the costs were kept the same for both the core and accelerated scenario in 2005 at 32.4 GBP.GJ^-1^, but then were linearly reduced in the accelerated scenario until 2020 to 25.6 GBP.GJ^-1 ^(DTI's low estimate) (Table 3). The capital costs were kept level from 2020 to 2050, although lower costs might be possible if feedstocks become cheaper (J. Rogers, unpublished data).

The efficiency of fast pyrolysis in the accelerated scenario did not change from the 90% found in the core scenario. Variable O&M costs were modelled in 2000 in both scenarios at 3 GBP.GJ^-1^. In the accelerated scenario, however, O&M costs were reduced after 2000 to represent a figure of 4% of the capital costs, dropping to 1 GBP.GJ^-1 ^by 2050 (to be consistent with the literature [22]).

### Bioengineering of energy plants

Acceleration of bioengineering of energy plants focused on domestic energy crops (within the UK) to reflect the environmental sustainability criteria. Imported energy crops were not accelerated. Improvements in the yield of energy crops are predicted to be the major factor that will accelerate the development of energy crops (Table 1), and therefore, future crop costs were based on a doubling of the average yield by 2050 and, to some extent, improvements in agro-machinery for growing and harvesting energy crops.

A literature review of energy crop costs highlighted the wide range of plants suitable as bioenergy crops. Data obtained for this scenario, however, focused only on those crops which are suitable to be grown in the UK (miscanthus, willow, switchgrass and poplar).

Although there are a wide variety of bioenergy crops with different crop costs, MARKAL uses an average figure to represent all energy crops. The estimates for crops from the literature e.g. [30-34] therefore were averaged to give one 'energy crop' cost, to be consistent with current methods used in the model. All costs found in these studies were converted into a comparable unit (GBP.GJ^-1^) using an assumption of average yield of 12 t.(ha.yr)^-1 ^increasing to a future yield of 24 t.(ha.yr)^-1 ^in 2050.

To model accelerated development of energy crops, costs for 2000 were kept the same as the core scenario at 3.61 GBP.GJ^-1^, but in the ATD scenario this was decreased to 2.9 GBP.GJ^-1 ^in 2010 and 1.45 GBP.GJ^-1 ^by 2050, to represent a gradual improvement in biotechnology from 2000 to 2050 (Table 3). As gradual improvements (rather than step changes) are expected, the costs were modelled as a linear decline between these capital cost points. In addition to reducing the crop costs, the predicted increase in yield would also increase the upper bounds of available crops (with higher yields, more crops can be grown on the same amount of land). Therefore, in the accelerated scenario, the upper bound of domestic energy crops available was doubled to reflect the doubling of yield.

### Agro-machinery

No new changes were made to the accelerated development dataset due to improvements in agro-machinery (Table 1). Improvements in agro-machinery are expected to be one of the factors influencing the declining costs of growing and harvesting energy crops. Accordingly, these improvements were included as a factor affecting the bioengineering of energy plants accelerated data, as both of these underpinning technologies are represented as one resource cost in MARKAL.

## Results

### Bioenergy penetration in the Bioenergy ATD scenario

Overall, there is a larger uptake of bioenergy in the ATD Bioenergy scenario than in the LC-Core scenario.

### Resources

**Figure 3:**
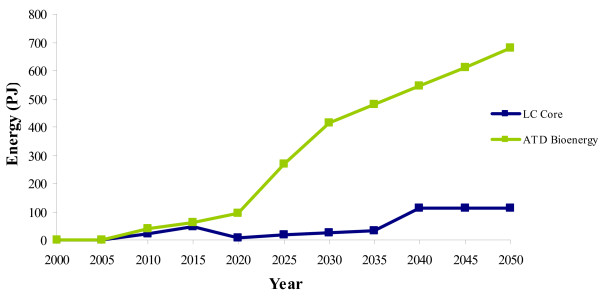
**Energy crop production**. LC-Core (blue) and the accelerated technology development (ATD) Bioenergy scenario (green).

The increased uptake of bioenergy in the ATD scenario appears to be due to the availability of cheaper resources (energy crops). Although energy crops are utilised in both scenarios in 2010, there is a much larger uptake of bioenergy crops across all vintages in the ATD scenario from 2010 to 2050 (Figure 3). The land available for energy crop production is not fully utilized in the LC-Core scenario and produces a maximum of 113 PJ of domestic energy crops. The production of energy crops in the ATD scenario, however, reaches a physical constraint when all available domestic land for energy crop production is utilized in 2030 (at 415 PJ). Energy crops continue to increase in terms of PJ, after 2030 in the ATD scenario due to the accelerated assumption of increasing yields. This allows for increased energy from energy crops on the same amount of land. Accordingly, in 2050, there are 679 PJ of energy crops in the ATD scenario (compared with 113 PJ in LC-Core).

### Electricity sector

**Figure 4:**
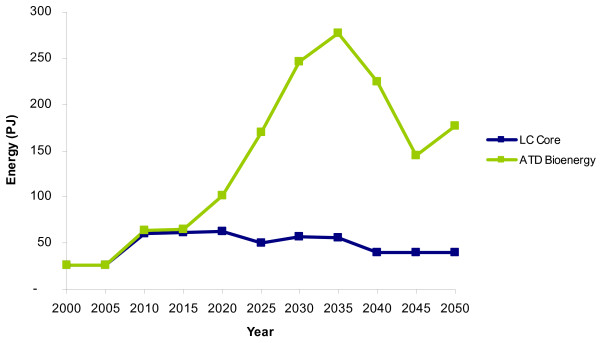
**Electricity produced from biomass**. LC-Core (blue) and the accelerated technology development (ATD) bioenergy scenario (green).

The production of electricity from biomass (primarily from gasification) reaches a peak of 277 PJ of electricity (roughly 19% of total electricity generation) in 2035 in the ATD scenario, compared with a peak of only 62 PJ in the LC-Core in 2025 (Figure 4). This increase in uptake is largely due to an increased adoption of gasification technologies in the ATD scenario. Gasification of solid biomass (energy crops) is first selected as a viable option for electricity generation in 2010 in both scenarios. However, high levels of energy crop gasification are deployed for electricity generation in the ATD scenario, whereas gasification is not deployed after 2010 in the LC-Core scenario.

**Figure 5:**
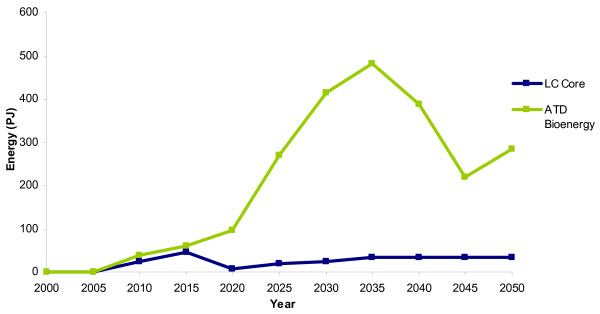
**The distribution of solid biomass for electricity production**. LC-Core (blue) and the accelerated technology development (ATD) bioenergy scenario (green).

In the ATD scenario, the use of solid biomass for electricity generation increases until 2040, when it reaches 481 PJ of energy crops. After 2040 the use of energy crops for electricity generation decreases, reaching 284 PJ by 2050 (Figure 5). This decline occurs because energy crops are shifted away from electricity production to be used for heating in the residential sector after 2040.

The deployment of gasification in the ATD Bioenergy scenario has an effect on the deployment of other electricity generation technologies. It takes significant market share from wind from 2015 to 2050 and from nuclear in the medium term (2025 to 2035).

The deployment of gasification in the ATD scenario also has some smaller effects on the levels of adoption of other bioenergy technologies. For instance, biomass district heating technologies (heat only) in the ATD Bioenergy scenario are used more than in the LC-Core scenario in the short term, but significantly less in the longer term. In addition, the deployment of gasification also means that biomass CHP plant (LTH) is never deployed in the ATD scenario whereas it was deployed from 2035 onwards in the LC-Core scenario.

### Residential/service sector

**Figure 6:**
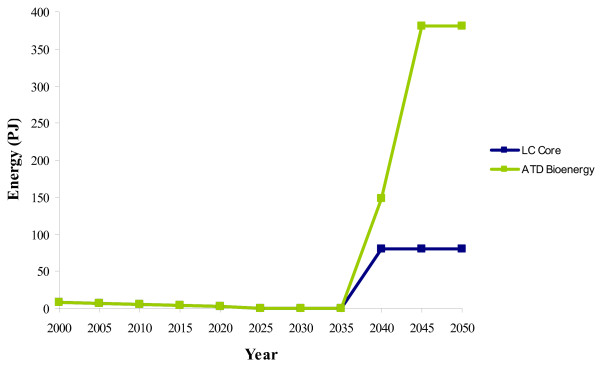
**The use of biomass in the residential heating sector**. LC-Core (blue) and the accelerated technology development (ATD) bioenergy scenario (green).

Accelerated technology development of bioenergy creates changes in the residential heating sector when compared with the LC-Core scenario (Figure 6). There is an uptake of solid biomass (from energy crops) in the residential/service sector in 2045 in both scenarios, but it is much higher in the ATD scenario. In the LC-Core scenario there are 80 PJ for residential heat by 2050 while in the ATD scenario there are 395 PJ by 2050.

In the service sector, woodchips are displaced by pellets (from energy crops) from 2040 onwards in the ATD scenario, unlike the LC-Core scenario where wood is used until 2050. The increasing use of energy crops for the residential and service sectors in the long term corresponds to the timing of the declining use of energy crops for electricity and the continuing increase in production of energy crops.

### Transport sector

**Figure 7:**
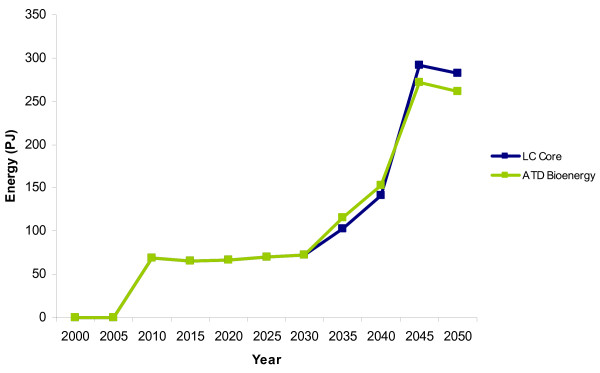
**Final energy demand for biofuels in the transport sector**. LC-Core (blue) and the accelerated technology development (ATD) bioenergy scenario (green).

Overall, final energy demand from biomass in the transport sector does not differ significantly between the LC-Core and ATD Bioenergy scenarios. The total transport fuel demand is the same in both scenarios until 2035 to 2040, when the total transport fuel demand in the ATD is slightly higher (152 PJ) than in the LC-Core (142 PJ) (Figure 7). However, by 2050 the transport fuel mix in the ATD scenario has more conventional transport fuels than the LC-Core. In the ATD scenario, there is less ethanol and more biodiesel, diesel and petrol than in the LC-Core scenario.

The overall impact of acceleration on ethanol is negative because less domestic ethanol is produced in the ATD scenario. Imported ethanol remains at similar levels in both scenarios. There are two pathways for the production of ethanol in MARKAL: traditional straw fermentation and lignocellulosic conversion to ethanol. In the ATD scenario, traditional ethanol from wheat straw fermentation is deployed later and at lower levels (170 PJ in LC-Core *vs*. 103 PJ in ATD Bioenergy). However, there is an increase in the uptake of the accelerated lignocellulosic ethanol in the ATD scenario from 2035 onwards (this technology was not deployed after 2035 in the LC-Core scenario). However, the increase in lignocellulosic ethanol is smaller than the decrease in traditional wheat ethanol and therefore there is an overall reduction in the level of ethanol in the ATD scenario.

The overall reduction in ethanol deployment and increase in conventional fuels in the ATD scenario suggests that under the least cost assumptions of the model and accelerated bioenergy assumptions, it is more economical to use biomass to decarbonise the electricity and residential heat sectors. As a result, in the accelerated scenario, there is more decarbonisation of electricity and heat and less need for more expensive transport sector decarbonisation.

### Bioenergy in the Aggregated Accelerated scenarios

#### LC Acctech (60%) no fuel cells

**Figure 8:**
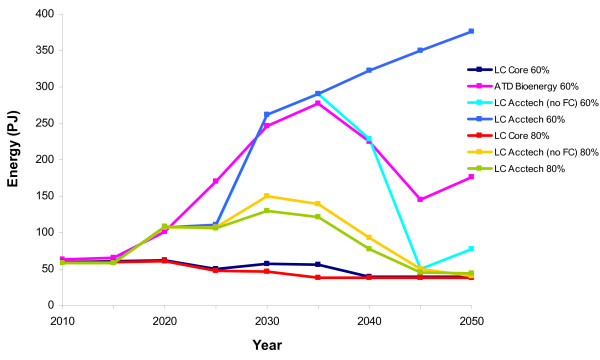
**Biomass for electricity production in the aggregate scenarios**. LC-Core (60%) (dark blue); accelerated technology development bioenergy (ATD Bioenergy (60%)) (purple); LC Acctech without fuel cells (60%) (LC Acctech (no FC) 60%) (aqua); LC Acctech with fuel cells (60%) (LC Acctech 60%) (blue); LC-Core (80%) (red); LC Acctech without fuel cells (80%) (LC Acctech (no FC) 80%) (yellow); LC Acctech with fuel cells (LC Acctech 80%) (green). Percentage value corresponds to carbon reduction targets.

**Figure 9:**
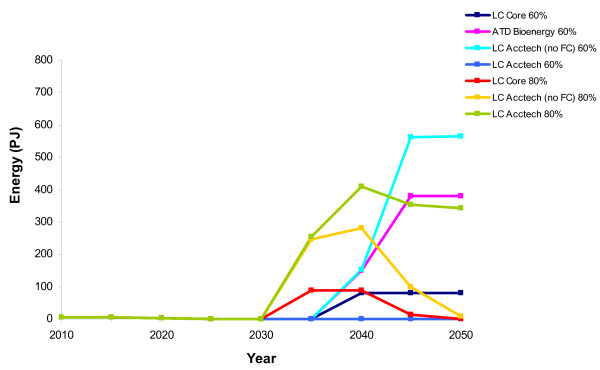
**Residential heat from biomass in the aggregate scenarios**. LC-Core (60%) (dark blue); accelerated technology development bioenergy (ATD Bioenergy (60%)) (purple); LC Acctech without fuel cells (60%) (LC Acctech (no FC) 60%) (aqua); LC Acctech with fuel cells (60%) (LC Acctech 60%) (blue); LC-Core (80%) (red); LC Acctech without fuel cells (80%) (LC Acctech (no FC) 80%) (yellow); LC Acctech with fuel cells (LC Acctech 80%)(green). Percentage value corresponds to carbon reduction targets.

**Figure 10:**
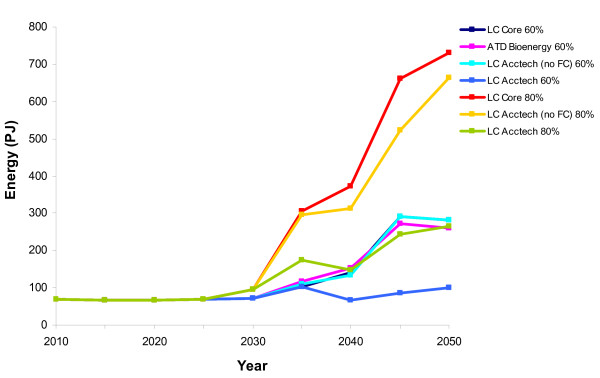
**Biomass for transport (biofuels) in the aggregate scenarios**. LC-Core (60%) (dark blue); accelerated technology development bioenergy (ATD Bioenergy (60%)) (purple); LC Acctech without fuel cells (60%) (LC Acctech (no FC) 60%) (aqua); LC Acctech with fuel cells (60%) (LC Acctech 60%) (blue); LC-Core (80%) (red); LC Acctech without fuel cells (80%) (LC Acctech (no FC) 80%) (yellow); LC Acctech with fuel cells (LC Acctech 80%) (green). Percentage value corresponds to carbon reduction targets.

When all the technologies are accelerated together in the LC Acctech (no fuel cells) scenario at 60% carbon reduction, there is less biomass for electricity after 2040 than there was in ATD Bioenergy (Figure 8). This is likely due to the abundance of other cheap alternatives for electricity production. However, there are dramatically higher levels of biomass being used for residential heat in LC Acctech (no fuel cells) (60%) than in ATD bioenergy (Figure 9). Whereas in 2050 there are 381 PJ of residential biomass in ATD Bioenergy there are 564 PJ in LC Acctech (no fuel cells) (60%). None of the other technologies accelerated in this scenario offers a competing low carbon option for heat and therefore more biomass is used for heat than electricity. Biomass for transport changes less noticeably between ATD Bioenergy and Acctech (no fuel cells) (60%) than it does for the electricity and heat sectors (Figure 10). However, there is a small increase in use of biomass for transport biofuels in LC Acctech (no fuel cells) (60%) as compared with Bioenergy ATD.

#### LC Acctech (80%) no fuel cells

When accelerating all the technologies together at an 80% carbon reduction, there are again major changes to the distribution of biomass. While there are still high levels of biomass being utilised in LC Acctech (no fuel cells) (80%), the biomass is being distributed to the sectors differently.

In LC Acctech (no fuel cells) (80%) there is much less biomass deployed for electricity production (a peak of 150 PJ as opposed to 290 PJ in LC Acctech (no fuel cells) (60%) (Figure 8). There is also a significant reduction in biomass deployed to residential heating (Figure 9). In 2050 in the 60% scenario, there are 564 PJ of biomass used in the residential sector, while in 2050 in the 80% scenario there are only 8 PJ used in this sector. While there are reductions in biomass for electricity and residential heat, there is a large increase in biomass for transport biofuels in the 80% scenario (Figure 10). There are 281 PJ of transport biofuels in the 60% scenario and 665 PJ in the 80% scenario. With a higher carbon reduction target, there is an increased utilisation of biomass for transport biofuels instead of heat and electricity in LC Acctech (no fuel cells) (80%).

#### LC Acctech (60%) with fuel cells

When all the technologies (including fuel cells) are accelerated for a 60% carbon reduction target then bioenergy is used more for electricity generation than in any other scenario (including the single technology acceleration-ATD Bioenergy) (Figure 8). However, biomass is not heavily used for heat or transport (Figures 9 and 10). In fact, from 2025 onwards there is no biomass used for residential heating and there is less biomass in the transport sector than there was even in the LC-Core scenario.

#### LC Acctech (80%) with fuel cells

When all the technologies (including fuel cells) are accelerated with an 80% carbon reduction target then biomass is used less for electricity generation than in the other accelerated scenarios (Figure 8). However, biomass is utilised more for heat than in any other 80% scenario in the later period (2035 onwards) (Figure 9). Transport biofuels are utilised more than they are in the ATD Bioenergy scenario (at 60%) but significantly less than in the other 80% scenario (Figure 10). This is likely due to the new availability of hydrogen transport options to decarbonise the transport sector.

## Discussion

This study highlights the potential for innovation throughout the bioenergy supply chain to contribute to the decarbonisation of multiple sectors of the UK energy system. Based on the research narratives developed and techno-economic modelling scenarios, the results suggest that bioenergy has the potential to be an affordable option to decarbonise not only the electricity and residential heat sectors, but also the transport sector under an 80% carbon reduction target given further technological development.

When bioenergy technologies are accelerated in isolation in the ATD Bioenergy scenario, electricity production from biomass is highly deployed in the medium term followed by increased residential heat from pellets (energy crops) in the long term. Until 2035, a similar pattern was seen for electricity generation from biomass in the aggregated scenario (LC Acctech (60%) with fuel cells) with a 60% carbon reduction. However, in the 60% aggregated scenario without fuel cells, there was less electricity production from 2040 to 2050 and much more biomass for residential heating than in the ATD Bioenergy scenario. This suggests that in the aggregated scenarios, electricity produced from bioenergy crops becomes less economically competitive than other accelerated low carbon electricity options such as marine, wind power and solar PV. The flexibility of bioenergy means it can be used in multiple end use sectors, while the other renewables cannot. Biomass therefore becomes better used as a low cost option to decarbonise residential heating in the aggregated scenarios when competing with other renewables.

Under the increased carbon reduction targets in the aggregated 80% scenario without fuel cells (LC Acctech (80%) no fuel cells scenario), the distribution of biomass changes. There is significantly less electricity and residential heat generated from biomass but more biomass in the transport sector (biofuels) in the 80% scenarios. The higher carbon reduction target of 80% results in more pressure on the transport sector to decarbonise compared with the 60% scenarios. Without fuel cell acceleration, there are few affordable options to decarbonise transport and biofuels is the cheapest option. Therefore the model shifts much of the biomass away from electricity and heat and towards transport biofuels. This suggests that with a limited resource like biomass there should be a thorough investigation into the optimal utilisation of the resource to decarbonise the economy.

When fuel cells, an alternative option for decarbonising the transport sector, are introduced in LC Acctech with fuel cells at a 60% or 80% carbon reduction, the fuel cells are deployed for decarbonisation of the transport sector. In the 60% scenario this leads to biomass being used primarily for electricity generation and heat while in the 80% scenario biomass is used earlier for residential heating. This reinforces the message that the optimal distribution of biomass depends on the ambition of the carbon target and the availability of alternative low carbon technologies. A whole system mentality must be used when determining how to best use biomass resources.

Given the importance of low cost biomass resources in the increased uptake of bioenergy in the ATD Bioenergy scenario, cheaper feedstocks are clearly important for the future deployment of bioenergy technologies. This suggests that much of the scope for accelerated deployment of bioenergy comes from the development of more efficient, low cost energy crops. This can be achieved through increasing the yield, crop resistance to disease and pest species and by increasing successful establishment of perennial species. Feedstock flexibility is also important for many of the bioenergy technologies, and therefore improvements in this area will increase the economic competitiveness of bioenergy.

The results from all the scenarios suggest that using biomass for residential heat is a potential option to decarbonise the UK's energy market when bioenergy is competing with other accelerated technologies under 60% carbon reduction targets. However, in 80% carbon reduction scenarios, transport biofuels are deployed at much higher rates. This suggests that to achieve a higher level of decarbonisation, transport will need to be highly decarbonised and that lignocellulosic ethanol could be one way to achieve this. Given the uncertainties surrounding the ATD figures and our 'what-if' rather than a predictive approach, however, it remains to be seen whether bioenergy technologies will develop and be deployed in this way.

Fast pyrolysis for bio-oil production was not deployed in any of the scenarios. This certainly does not mean that fast pyrolysis technology is without potential. To fully understand why fast pyrolysis was not deployed, a sensitivity analysis would need to be undertaken on the costs of pyrolysis technology within the model; however, due to time constraints this was not possible. It is additionally important to highlight that the model may not capture some key advantages in using fast pyrolysis in an energy system which are beyond cost competitiveness. MARKAL is used for 'what-if' analysis and focuses on 'least-cost' solutions for the energy system over the time horizon chosen. Consequently, MARKAL will select the technologies which supply energy at the lowest cost, even if this only represents a marginal cost saving. One of the consequences of this modelling paradigm is that some technologies/pathways may not be selected by the model as part of the 'optimal' energy system configuration even if in reality they could be developed. In addition the MARKAL modelling framework can only capture some of the 'non-economic' benefits of certain energy technologies/pathways, which influences its choice of 'solutions'. Although the UKERC 2050 MARKAL model has, additionally, been thoroughly tested (and has been constructed from earlier also tested versions of the UK-MARKAL model), it has not been built specifically to explore bioenergy pathways. Within the time constraints of the project, it was not possible to improve the bioenergy chains represented within the model. Within the TSEC-BIOSYS modelling exercise, however, bioenergy chains were specifically improved. 'Domestic' fast pyrolysis also was not deployed in the system; however, imported bio-oil was deployed most notably in the industrial sector within the TSEC-BIOSYS model (unpublished data). The 'imported' bio-oil pathway is currently not modelled within the MARKAL model used for UKERC 2050 and this highlights an area where the model needs to be improved.

Land availability within the UK for growing bioenergy crops is, additionally, a big issue within the bioenergy field. In MARKAL, the upper bounds of available energy crops were capped to reflect the limited availability of land for biomass in the UK. However, there are also other issues associated with bioenergy that could further limit biomass levels in the UK. Bioenergy is often considered controversial due to issues surrounding direct and indirect land use change [13,35], real carbon reduction potential, social acceptability and other environmental impacts. There are many concerns about the sustainability of using first generation food crops for energy production due to possible impacts on food prices and increased and/or accelerated land-use change. As a result, there is a great deal of research and support for second generation dedicated energy crops, which do not compete with food crops, do not negatively affect the quality of the land and do not negatively shift the pattern of land use (for example that do not require clearing certain types of land to grow energy crops). The Gallagher Review recommended that bioenergy crops should be grown on marginal or idle land, and research in this area will become important for the future of bioenergy deployment [13]. These socio-environmental limits are not represented in the model and thus would impose additional deployment constraints not shown in the modelling results. This highlights some limitations of cost optimisation models. The modelling overlooks many barriers to development and deployment of technology other than costs, including some key aspects relating to both the spatial and the temporal infrastructure of bioenergy. It is also very important that the modelling work is underpinned by clear qualitative stories, including policy implications.

A further key factor in determining the use of UK land for energy crop production will be the availability and price of imported biomass. This is important given that at least half of the current biomass supply is sourced from outside the UK [4] and that domestic bioenergy crops are struggling to be adopted by UK farmers. UK growers appear reluctant to diversify into unfamiliar perennial crops which are associated with long contracts with energy supply companies. This has been exacerbated by the recent uncertainties over support for the Energy Crops Scheme [36]. The reliance on imported biomass also has implications for long-term environmental sustainability of bioenergy technologies. The influence of imported biomass on bioenergy deployment, however, could possibly be explored in future MARKAL runs.

### Limitations

Social and environmental limitations on bioenergy development and deployment, such as the wide-scale environmental costs and benefits of bioenergy deployment on ecosystem services, and direct and indirect land use displacement, may make deployment of bioenergy technologies challenging. Overall, however, the work suggests that bioenergy can contribute significantly to a low carbon UK energy future. However, it is important to keep in mind that (1) the modelling overlooks many barriers to development and deployment other than costs; (2) the modelling does not properly model some key aspects linked to bioenergy infrastructure, both spatial and temporal, and (3) the figures used in the ATD scenarios are uncertain and should be taken with caution. This work offers insights into the potential accelerated technology development and deployment that could occur along selected bioenergy chains in the UK. It should be taken as an illustration, rather than a prediction, of how bioenergy could be deployed in the future UK energy system. The analysis undertaken contributes mostly from the illustration of 'what could be done' with MARKAL to look into the potential of ATD and bioenergy.

Our findings are limited by the uncertainty on the values chosen to represent future ATD. For example, the results for the deployment of lignocellulosic ethanol are based on very high conversion efficiencies, and should be taken with caution, and not as a prediction of the capability of lignocellulosic technology deployment. Rather, the results from this should be looked at as an illustration of the capability of the model, and as highlighting where further work is needed. The UK-MARKAL database is iteratively constructed and improved, and this has been corrected for further runs. However, as a consequence of time constraints, it was not possible to include this revised value for the ATD runs for the present work.

In addition, the focus was mostly on one scenario. A single ATD Bioenergy scenario was modelled in MARKAL which combined the accelerated development input data of the five bioenergy chains that were selected for exploration. Although the use of different scenarios would help to account for some of the uncertainties in the figures used in the scenarios, this was not possible given the time constraints of the project. In future, these uncertainties would need to be taken into account, for instance, by using different scenarios, including the use of more bioenergy chains, and by undertaking sensitivity analyses to determine which parameters are most influential on the deployment of the bioenergy technologies explored.

Moreover, the scenarios focused on five select technologies/bioenergy chains. There are other promising bioenergy technologies which have the potential to be economically viable and sustainable, especially those where active research is being conducted both in the UK and internationally. Some technologies, like algal fuels, have potential but are currently not represented within MARKAL.

It is also important to highlight that the failure of a technology to be deployed in these scenarios does not mean that technology is without potential. As MARKAL is a least-cost optimisation model, it will select the cheapest technology to serve the demand while satisfying the constraints, even if those cost savings are only marginal.

Although the UKERC 2050 MARKAL model has, additionally, been thoroughly tested (and has been constructed from earlier also tested versions of the UK-MARKAL model), it has not been built specifically to explore bioenergy pathways. Consequently, this means our approach is challenging, but innovative nonetheless. Here the authors have illustrated the possibilities of the model, but within the time constraints of the project, it was not possible to improve the bioenergy chains represented within the model. The 'imported' bio-oil pathway is currently not modelled within the UKERC 2050 model and this highlights an area where the model needs to be improved.

The costs used in the modelling were additionally calibrated on a 2000 base year and we are conscious of the limitations of this approach and the possibility of improvement to represent more carefully the 'short term'. The value of the modelling exercise, however, may be more in exploring the longer term trends.

Further work is needed to build on our proposed approach and it could be useful to systematically look at the pathways to answer the question 'how much improvement is necessary in the different biotechnologies before they can be expected to have a significant role in the future energy system?'

This paper has highlighted the applicability of an original modelling approach, but future work should be focused to address some of the above limitations. It is also important to note that the modelling has been underpinned by clear qualitative stories, including R&D directions and potential and policy implications, which give the modelling results context. The UK-MARKAL model is an 'iteratively built' database and model and by highlighting its possibilities as well as current gaps in data representation and/or capabilities, we continue to contribute to the future improvements of the model.

## Conclusion

This work explores the potential of bioenergy technologies to contribute to carbon reductions in the UK energy system through accelerated technology development. The analysis undertaken represents an illustration of 'what-if' scenarios in MARKAL to explore accelerated technology development of bioenergy technologies.

The exercise has highlighted that there is much potential for accelerated technology development in the five bioenergy technologies investigated in this paper, particularly in bio-engineering of energy crops as it underpins many bioenergy chains. Given further development, bioenergy technologies could become increasingly more economically competitive with fossil-based technologies as feedstock costs are reduced in line with crop improvements due to plant breeding efforts, the ability to grow energy crops on marginal lands, increased crop resistance to disease and pests, cheaper enzymes for lignocellulosic conversion to bio-ethanol, and improvements in gasification and fast pyrolysis technologies. There is additional potential for advances in other bioenergy technologies not assessed in this paper, which could help to drive the commercial availability and competitiveness of bioenergy technologies in the R&D stage further forward.

This paper highlights the unique flexibility of bioenergy technologies to potentially decarbonise multiple sectors. Under all the scenarios there was a high deployment of bioenergy, which implies that it is possible that bioenergy will be a valuable part of the pathway to a decarbonised economy. It is important to highlight, however, that figures used in the ATD scenario were uncertain and should be taken as an illustration of how much improvement is needed in the five technologies for the levels of market penetration seen in the model output. Interestingly, carbon reduction targets influenced the bioenergy mix deployed in the UK energy market. Lower targets (60%) resulted in more electricity and residential heat, while higher targets (80%) resulted in increased deployment of biomass for biofuels. Acceleration of bioenergy without other technologies accelerated, however, led to more electricity from biomass because other low carbon electricity options were less cost competitive.

Innovation at all stages of the bioenergy supply chain is important and can contribute to increased chance of deployment. Future R&D efforts and innovation are therefore essential at all points along the supply chain.

Ultimately, the future deployment of bioenergy technologies is dependent on many different factors including investment and R&D efforts, carbon reduction targets and the ability to compete with other low carbon technologies as they become deployed.

## Abbreviations

ATD: accelerated technological development; BERR: Department for Business, Enterprise and Regulatory Reform; CCC: Committee on Climate Change; DTI: Department of Trade and Industry; GBP: pounds sterling; NREL: National Renewable Energy Laboratory; O&M: operating and maintenance; R&D: research and development; RD&D: research, development and demonstration; UKERC: UK Energy Research Centre.

## Competing interests

The authors declare that they have no competing interests.

## Authors' contributions

DC collected quantitative and qualitative data for most of the chains, interpreted the results and drafted the manuscript. SJ collected quantitative and qualitative data for the lignocellulosic chain, and helped to draft the manuscript. BM helped with data collection, interpreted the results and also drafted the manuscript. GA undertook the modeling work and commented on the manuscript. GT helped draft the manuscript. All authors read and approved the final manuscript.
